# Formation of Homogeneous Nanostructure via Interference of Square Flattop Femtosecond Laser Pulses

**DOI:** 10.3390/nano15050355

**Published:** 2025-02-25

**Authors:** Takemasa Sumimoto, Godai Miyaji

**Affiliations:** Faculty of Engineering, Tokyo University of Agriculture and Technology, 2-24-16, Nakacho, Koganei 184-8588, Tokyo, Japan

**Keywords:** femtosecond laser processing, LIPSS, nanostructure formation, beam shaping, flattop beam

## Abstract

We report on the formation of homogeneous nanostructures using a two-step ablation process with square flattop beams of femtosecond (fs) laser pulses. The Gaussian beam output from a ytterbium fs laser system was converted to a square flattop beam by a refractive beam shaper and a square mask. This beam was split into two with a diffraction optical element, and then the downsized beams were spatially and temporally superimposed on a titanium surface. In the first step, the interference fringes of these two beams formed grooves with a period of 1.9 µm through ablation. Next, the surface was irradiated at normal incidence by a single beam to form a homogeneous line-like nanostructure with a period of 490 nm in a 53 μm square area. This nanostructure had a constant period and was formed over 95% of the laser-processed area, indicating that the ratio between the nanostructure and modification area was over six times larger than that for a Gaussian beam.

## 1. Introduction

In recent years, the demand for nanofabrication technologies has surged across various fields of industry, particularly in the manufacturing processes of semiconductor-based optical and electronic devices. These processes typically involve a combination of lithographic techniques, etching processes [1,2,3], and nanoimprinting [4,5]. However, traditional lithography requires numerous chemical materials and multiple steps, while nanoimprinting is challenging to apply to hard materials. Additionally, while electron beam lithography offers high spatial resolution, it can only process a limited area in a given time frame compared to photolithography.

Laser beams have presented an alternative for fast material processing in air without physical contact. They can also modify the interior of transparent materials. Ultrashort laser pulses with femtosecond (fs) to picosecond durations allow high-quality processing with sharp edges via non-thermal interactions [6]. This capability has made ultrashort laser pulses increasingly popular in precision microfabrication technology, such as creating vias in semiconductor device substrates [7] and periodic structures on solid surfaces using the interference fringes of fs laser pulses [8,9,10,11,12].

Moreover, intense fs laser pulses can produce periodic structures on solid surfaces with periods much smaller than the laser wavelength (tens to hundreds of nanometers) [13,14,15,16,17,18,19,20]. These are referred to as laser-induced periodic surface structures (LIPSSs). Various properties of surfaces having such nanostructures can be controlled, including wettability [21,22,23,24], light absorption [25], light phase and polarization [26], surface coloration [22,27], friction coefficient reduction [28], and cell spreading direction [29]. This manufacturing technique has thus gained attention as a simple, waste-free approach to nanofabrication. However, the size and shape of the resulting nanostructures are inhomogeneous in an ablation trace and depend on numerous factors, such as the material’s dielectric constant and surface condition, pulse duration, polarization, and pulse number. In particular, they are very sensitive to laser fluence and intensity [30].

To form homogenous nanostructures, Miyazaki and Miyaji developed a two-step ablation method [31]. This method can form homogeneous line-like nanostructures by irradiating a surface with fs laser pulses a second time after periodic microgrooves are first formed by the interference fringes of two-beam fs laser pulses. The period of homogeneous nanostructures is adjustable by the period of the microgrooves within a range of periods of the nanostructures that can be potentially formed by a single beam [31,32]. However, because the period of the nanostructure strongly depends on the laser fluence, the fs laser pulses, which have a Gaussian intensity distribution, can change the period in the laser spot.

In this paper, we demonstrate that a line-like nanostructure with a constant period over the whole area of the ablation trace can be formed using fs laser pulses with a constant intensity in the square region, also called a square flattop (SF) beam, and the two-step ablation process. The refractive-type field-mapping beam shaper converted a Gaussian beam output from the fs laser system into a tophat beam, and a metal mask shaped this into a square flattop beam. We show that the two-step method with the beam formed line-like nanostructures on a titanium surface with a constant period of 490 nm in a 53 µm square region. The area of the nanostructured surface with a constant period was 95% of the ablated or modified area, which was over six times larger than that formed with a Gaussian beam.

## 2. Experimental Setup

**Figure 1:**
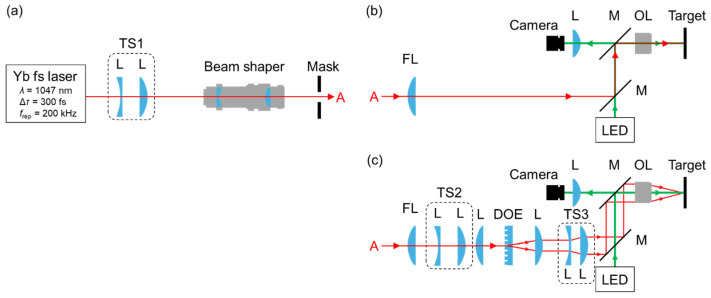
Schematic illustration of optical systems for (**a**) converting a Gaussian beam to a square flattop (SF) beam, (**b**) transferring a downsized image of the SF beam through a mask onto a target, and (**c**) interfering and transferring the images of SF beams onto the target. The character “A” denotes that the optical path is continuous. L: lens, TS: telescope, FL: field lens, M: dielectric mirror, OL: objective lens, LED: light-emitting diode, and DOE: diffractive optical element.

Figure 1 shows a schematic diagram of the optical configuration for the ablation experiment. We used linearly polarized, TEM_00_ mode laser pulses of a pulse duration Δ*τ* of 300 fs at a wavelength *λ* of approximately 1047 nm from a Yb laser amplifier system (Sprit 1040-30-HE, MKS Instruments, Inc., Andover, MA, USA) operated at a repetition rate *f*_rep_ of 200 kHz. The maximum pulse energy was 130 µJ, the beam diameter was 2.7 mm at 1/*e*^2^ intensity, and the M-squared value was 1.1. Firstly, as shown in Figure 1a, the beam diameter of the fs pulse was enlarged to 14 mm with a telescope (TS1) consisting of plano-concave and plano-convex lenses with focal lengths of *f* = −75 mm and 450 mm, respectively, and then converted to a tophat beam, which had a constant intensity in a circular area, with a refractive field-mapping beam shaper (πshaper 12_12_TiS_HP, AdlOptica Optical Systems GmbH, Berlin, Germany). An aluminum plate with a square aperture inserted just after the beam worked as a mask to cut out a square flattop beam, which had a constant intensity in a square area.

There are two types of beam shapers: a near-field mapping type [33,34,35,36,37] and a far-field type [38,39,40,41,42,43,44]. In terms of shaping principles, refraction [33,34,35,36,37,41,43,44] or diffraction [38,39,40,42] is generally used. Recently, a spatial light modulator has been developed, allowing for spatial and temporal control of arbitrary amplitude and phase [39,40,42]. In this study, we used the refractive near-field mapping shaper because it has higher energy efficiency than the diffraction type, and the spatial intensity distribution can easily be modified by the masking shape. Moreover, the beam shaper was implemented with simple optics consisting of only two aspheric lenses [36].

Next, as shown in Figure 1b, a plano-convex lens with *f* = 1500 mm was placed at a distance of 10 mm from the mask as a field lens (FL) to suppress beam divergence. The downsized image at the mask was transferred to the target surface at a magnification of 1/60 with an objective lens (OL, numerical aperture (NA) 0.25, magnification 10, *f* = 16.6 mm, pupil diameter 8 mm, OBL-10-A, SIGMAKOKI CO. LTD., Saitama, Japan). Here, the distance between the FL and OL was 970 mm. To observe the spatial intensity distribution of the SF beam at the target surface and the target surface, two dielectric multilayer mirrors (M) were placed between FL and OL, and the magnified image on the target was transferred onto a CMOS camera (CS165MU1/M, Thorlabs Inc., Newton, NJ, USA) with OL and a plano-convex lens of *f* = 150 mm. The output light of a light-emitting diode (LED, wavelength 660 nm, M660L4, Thorlabs Inc., Newton, NJ, USA) was used for illuminating the target surface.

Figure 1c shows the schematic diagram of the optical arrangement for interfering with the two SF beams at the target surface. First, the SF beam was magnified with TS2 consisting of *f* = −200 mm and 400 mm plano-concave and plano-convex lenses placed at a distance of 760 mm from FL. An achromatic lens of *f* = 75 mm was placed at a distance of 100 mm from TS2, and a diffractive optical element (DOE, DS-033-800-Y-A, HOLO/OR Ltd., Ness Ziona, Israel) was placed at a distance of 75 mm from this achromatic lens. This achromatic lens transferred the downsized image at the mask onto the DOE at a magnification of 1/27. The DOE could generate first-order diffraction light at an angle of 4.9 degrees from the optical axis, and its diffraction efficiency was 30% per beam. Finally, an aspheric lens of *f* = 25 mm and a telescope (TS3) consisting of plano-concave and plano-convex lenses of *f* = −150 mm and 200 mm were placed between the DOE and M to transfer the image on the DOE onto the target with a magnification of 1/3.0. The interval between the two beams just before the OL (NA 0.4, magnification 20, *f* = 9.01 mm, pupil diameter 7.98 mm, OBL-20-A, SIGMAKOKI CO. LTD., Saitama, Japan) was 5.1 mm. Here, the reason why the combination of the DOE and an afocal image transferring system was used for two-beam interference was that the peak position of the fs pulse needed to be parallel to the target surface to form an interference area in the entire beam spot [10]. Furthermore, the interfering system using DOE is beneficial and easy to handle because the two beams reach the target surface with equal optical path lengths, meaning that an optical delay stage is not needed [11,12].

A polished titanium (Ti) substrate with a surface roughness of *R*_a_ = 2 nm was used as the target for irradiation. The laser-irradiated surfaces were observed by scanning electron microscopy (SEM, JSM-6510, JEOL Ltd., Tokyo, Japan) and scanning probe microscopy (SPM, SPM-9700, SHIMADZU, Kyoto, Japan). The power density *S*(*u*, *v*) of the spatial frequency spectrum of the nanostructured surface was obtained by the two-dimensional (2D) Fourier transform of SEM images, where u and v are the spatial frequencies along the horizontal (*x*) and vertical (*y*) directions, respectively.

## 3. Results and Discussion

### 3.1. Intensity Distribution of SF Beam

**Figure 2:**
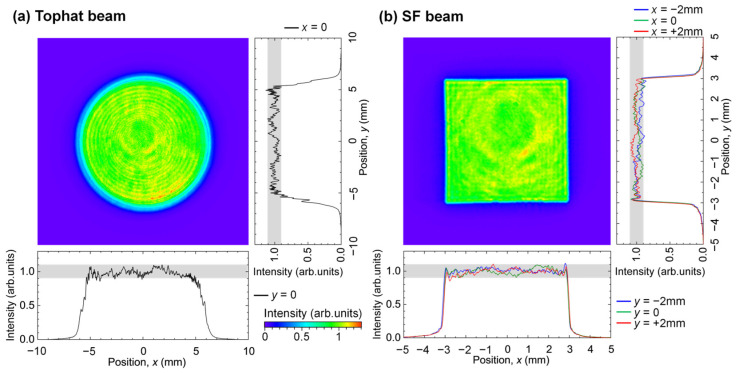
Intensity distributions of (**a**) tophat and (**b**) SF beams just after the beam shaper. They are normalized by the intensity averaged in beam areas. The gray in the intensity profile denotes the region of the intensity of 0.9–1.1.

Figure 2 shows the beam patterns at a distance of 10 mm from the beam shaper. Without inserting the mask, a tophat beam with a diameter of 12 mm was output, as shown in Figure 2a. Here, the intensity distribution was normalized by the intensity averaged in this circular region. As seen in Figure 2a, the intensity of the tophat beam was in the range of 0.9–1.1. Next, inserting the mask with a square aperture measuring 6.0 mm along the sides, an SF beam with 6.0 mm sides was obtained, as shown in Figure 2b. Here, the intensity distribution normalized by the intensity averaged in this square region falls within the range of 0.9–1.1 for the entire beam.

**Figure 3:**
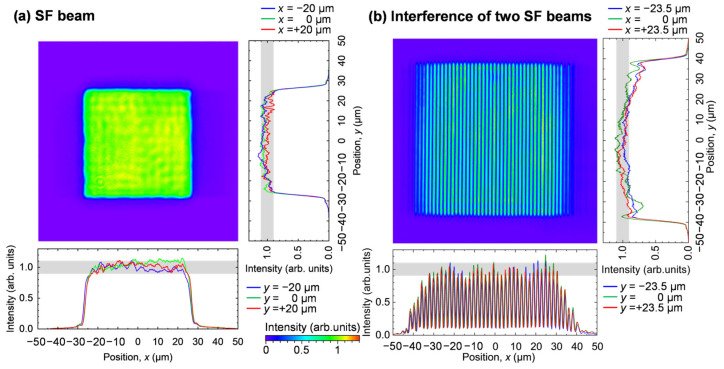
Intensity distribution on the target. (**a**) A single SF beam and (**b**) interference of two SF beams. The distributions are normalized by the intensity averaged in beam areas. The gray in the intensity profile denotes the region of the intensity of 0.9–1.1.

Figure 3 shows the intensity distribution of the SF beam at the target surface. Inserting the mask with a square aperture of 3.0 mm along the sides, a single SF beam had a constant intensity in a square region with sides of *a* = 50 μm, as shown in Figure 3a. Here, the intensity distribution is normalized by the intensity averaged in this square region. The intensity of the SF beam falls in the range of 0.9–1.1 with a steep rise in intensity from 0.1 to 0.9 at the 4.0 μm edges of the SF beam. This rise depends on the NA of the OL, which is due to the diffraction limit of light. Next, using the optical arrangement shown in Figure 1c and the mask with a square aperture of 6.0 mm sides, two SF beams were spatially and temporally superimposed on the target to form interference fringes. As shown in Figure 3b, interference fringes with uniform brightness and darkness were obtained in a rectangular region of 68 μm in the horizontal direction and 74 μm in the vertical direction. Each SF beam was incident on the target surface at an angle of *θ* = 16 deg. The period of the interference fringes was 1.9 μm, the bright intensity of the interference fringes was 1.0, and the dark intensity was 0.11. The two plane waves with *p*-polarization and *λ* incident oppositely at an incidence angle *θ* make fringes with a period of *Λ* = *λ*/(2sin*θ*) due to the superposition of the optical electric fields. The intensity distribution along the *x* direction is written as

(1) $$I\left(x\right)=I_{0}\left(1+\cos\left(\frac{4\pi }{\lambda }x\sin\theta \right)\cos\left(2\theta \right)\right),$$

where *I*_0_ is a constant value [45]. From Equation (1), *Λ* is calculated to be 1.9 μm, and if the bright intensity of the fringes is 1.0, the dark intensity is 0.10, so the experimental results agree with this theoretical value.

### 3.2. Nanostructure Formation by Single SF Beam

**Figure 4:**
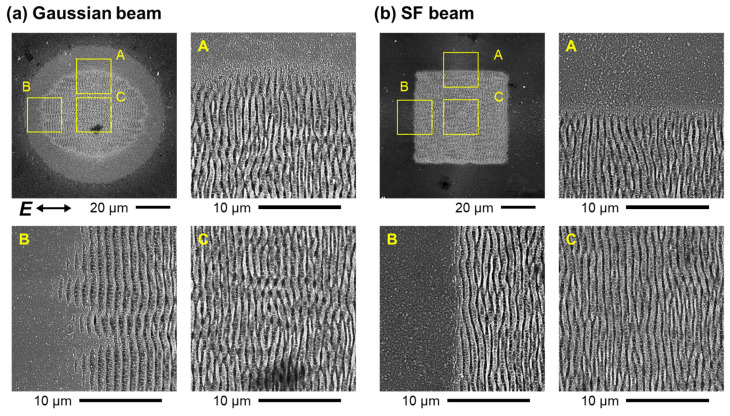
SEM images of Ti surfaces irradiated with fs pulses of (**a**) a Gaussian beam at *F*_0_ = 250 mJ/cm^2^, *N* = 50 and (**b**) an SF beam at *F* = 160 mJ/cm^2^, *N* = 50. The yellow square indicates the magnified observation area. A, B, and C represent the magnified SEM images at the upper edge, left edge, and center part of the ablation traces, respectively.

First, we observed Ti surfaces irradiated with a Gaussian beam, shown in Figure 4a. In the ablation experiment, the output fs pulses from the laser system were not passed through the beam shaper and were focused onto the target surface with a plano-convex lens of *f* = 150 mm instead of an objective lens. The diameter of the focal spot was 2*r* = 84 μm at 1/*e*^2^ intensity, where *r* denotes the radius. The fs pulses with *N* = 50 pulses at a peak fluence *F*_0_ = 250 mJ/cm^2^ formed nanostructures in an elliptical region of 62 μm and 47 μm in the center of the ablation trace. As seen in the enlarged SEM image, the nanostructure gradually disappeared within 5–10 μm of the edge of the elliptical region. In addition, surface modification was observed in the surrounding region with a radius of 42 μm from the center of the ablation trace. Here, the peak fluence *F*_0_ was defined as *F*_0_ = 2*U*_pulse_/(π*r*^2^), where *U*_pulse_ is the pulse energy of the fs laser.

Next, we observed the surfaces irradiated with the SF beam. The results are shown in Figure 4b. The fs pulses of the SF beam with *N* = 50 pulses and a fluence *F* = 160 mJ/cm^2^ formed nanostructures on the surfaces in a square region of 51 μm sides. As seen in the enlarged SEM image, the nanostructure gradually disappeared within approximately 2 μm of the edge of the nanostructured area. While debris was observed, no surface modification was found around the nanostructured area. The fluence *F* was defined as *F* = *U*_pulse_/*a*^2^ and *a* = 50 μm, as shown in Figure 3a.

**Figure 5:**
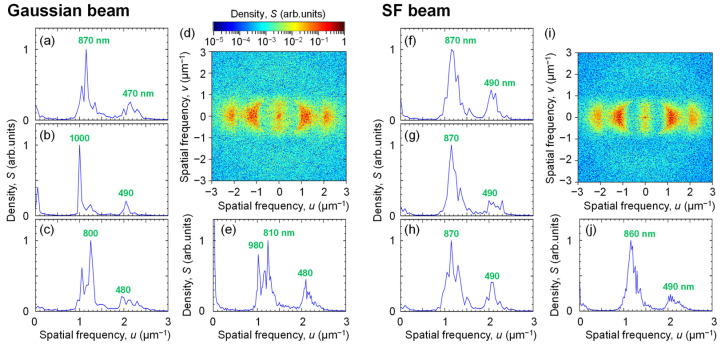
Notably, 2D spatial frequency spectral power density *S*(*u*, *v*) and its profile along the *x* direction *S*(*u*) of SEM image of Ti surfaces irradiated with fs pulses of (**a**–**e**) a Gaussian beam at *F*_0_ = 250 mJ/cm^2^, *N* = 50 and (**f**–**j**) an SF beam at *F* = 160 mJ/cm^2^, *N* = 50. *S*(*u*) in the 20 μm square region at (**a**,**f**) upper edge, (**b**,**g**) left edge, and (**c**,**h**) central part of the nanostructured surface. (**d**,**i**) *S*(*u*, *v*) in the 50 μm square region in the center of the nanostructured surface and (**e**,**j**) its *S*(*u*). The green number in *S*(*u*) denotes a period of the nanostructure at the peak.

To investigate the variance of the nanostructure periodicity with respect to the surface position, we calculated the spatial frequency spectra in the nanostructured surfaces. Figure 5a–c show the spatial frequency spectra *S*(*u*) along the *x* direction in the upper edge, left edge, and center areas in 20 µm square regions of the surfaces irradiated with the Gaussian beam, respectively. There are broad peaks at 1.15 µm^−1^ (870 nm) and 2.13 µm^−1^ (470 nm) on the upper edge, 1.00 µm^−1^ (1000 nm) and 2.04 µm^−1^ (490 nm) on the left edge, and 1.25 µm^−1^ (800 nm) and 2.08 µm^−1^ (480 nm) in the center. The position of the peaks is seen to vary with the position along the surface. Figure 5d,e show the 2D spatial frequency spectrum *S*(*u*, *v*) and its profile *S*(*u*) in a 50 µm square region in the center of the nanostructured surface, respectively. Broad and multiple peaks are seen at 1.02–1.23 µm^−1^ (810–980 nm) and 2.08 µm^−1^ (480 nm), and the broad spectrum is also distributed along the y direction, indicating that the linearity of the nanostructure is low and that the spatial frequency is not a single. The results indicate that the period of the nanostructure depends on fluence [30]. The area fraction of the nanostructured surface to the processed surface, including the modified region, was 41%. Especially of note, the area fraction of the nanostructured surface with constant periods of 480 nm and 800 nm to the processed surface was 16% in the circle with a radius of 17 µm in the center of the processed area.

Next, we investigated the spatial frequency spectrum of the surface irradiated with the SF beam. Figure 5f–h show *S*(*u*) in the upper edge, left edge, and center areas in 20 µm square regions of the nanostructured surfaces with the SF beams, respectively. The broad peaks at 1.15 µm^−1^ (870 nm) and 2.04 µm^−1^ (490 nm) do not change with the surface position. Figure 5i,j show *S*(*u*, *v*) and *S*(*u*) in a 50 µm square region in the center part of the nanostructured surface, respectively. The broad spectrum with peaks at 1.16 µm^−1^ (860 nm) and 2.04 µm^−1^ (490 nm) is also distributed along the *y* axis, indicating low linearity and no single spatial frequency component in the nanostructured surfaces. The area fraction of the nanostructured surface with a constant period to the processed surface was 95%, which was improved by approximately 6 times compared to that by the Gaussian beam.

### 3.3. Homogeneous Nanostructure Formation by SF Beam and Two-Step Ablation Method

**Figure 6:**
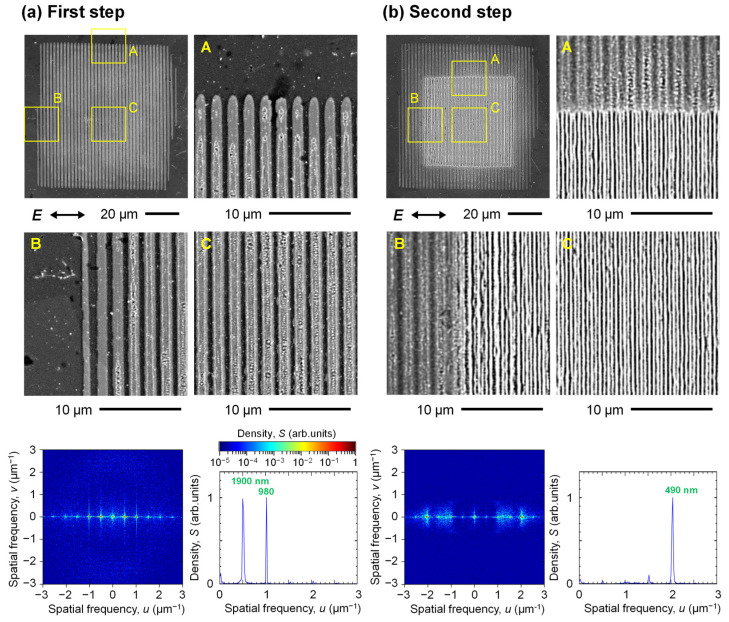
SEM images of Ti surfaces irradiated with fs pulses of (**a**) an interfered SF beam at *F* = 63 mJ/cm^2^, *N* = 3 per beam and (**b**) an SF beam at *F* = 110 mJ/cm^2^, *N* = 25 at normal incidence and their spatial frequency spectra. The yellow squares indicate the magnified observation areas. A, B, and C represent the magnified SEM images at the upper side, left side, and center part of the ablation traces, respectively. The green number denotes a period at the peak.

Using the SF beam and two-step ablation method, we formed homogeneous nanostructures on the surface. The results are shown in Figure 6. First, the surface was irradiated with the interference fringes of two SF beams shown in Figure 3b. Here, the fluence *F* = 63 mJ/cm^2^ per beam, and the pulse number *N* = 3. As shown in Figure 6a, an interference pattern with a period of 1.9 μm was formed in a rectangular region of 75 μm in the horizontal direction and 80 μm in the vertical direction. The spatial frequency spectrum was calculated from the SEM image of this rectangular region. The spectrum with a single spatial frequency is very sharp and narrow in the *x* and *y* directions, indicating a single period and good linearity of the microgrooves. Next, the single SF beam shown in Figure 3a was irradiated at normal incidence on the surface, with the interference pattern already formed, at *F* = 110 mJ/cm^2^, *N* = 25. A distinct homogeneous nanostructure was formed in a square region with 53 μm sides, as shown in Figure 6b. The spatial frequency spectrum calculated from the SEM image in this square region shows that a very sharp and narrow spectrum in the *x* and *y* direction of the nanostructured surface has a single spatial frequency at 2.04 µm^−1^ (490 nm), indicating that a homogeneous line-like nanostructure with good linearity and periodicity. As shown in. Figure 5f,j, the period of the nanostructure formed by the single-step fs laser irradiation had fixed ranges at the centers of 490 nm and 860 nm. On the other hand, the period of the homogeneous nanostructure by the two-step ablation method was an integer fraction of the interference pattern period within the range [31,32]. The results clearly demonstrate that surface plasmon polaritons of a single spatial mode were excited by the interference pattern formed on the Ti surface to form a homogeneous line-like nanostructure [31]. In the two-step ablation process, the fluence and pulse number of the fs pulse in the second step needed to be properly adjusted because the surface morphological change was very sensitive to them. The period of the homogeneous nanostructure shown in this paper was 1/4 of the interference pattern period. As with the single-step nanostructure formation, since increasing the fluence increased the nanostructure period, the period of the homogeneous nanostructure formed after the second step was easily 1/3 or 1/2 of the interference pattern period by increasing the fluence. In addition, the excessive pulse number of the fs pulses reduced the linearity and periodicity of the nanostructure. That is due to the ablation induced by the intense near-fields generated around the surface roughness, and too many pulses will disrupt the shape from the regular and homogeneous microgrooves formed by the first step.

**Figure 7:**
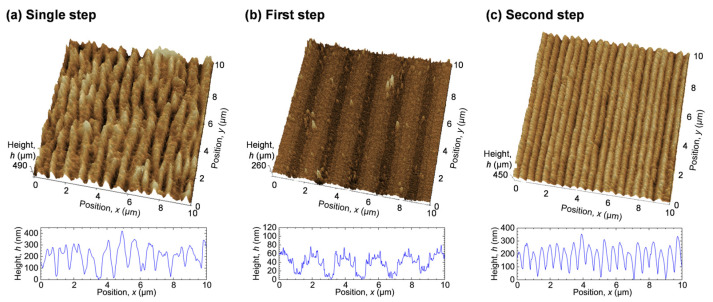
SPM images of Ti surfaces irradiated with fs pulses of (**a**) an SF beam at *F* = 160 mJ/cm^2^, *N* = 50, (**b**) an interfered SF beam at *F* = 63 mJ/cm^2^, *N* = 3 per beam, and (**c**) microgrooved Ti surface irradiated with fs pulses of an SF beam at *F* = 110 mJ/cm^2^, *N* = 25 at normal incidence.

Figure 7 shows SPM images of these surfaces. To compare with the nanostructure formed by the single-step fs laser irradiation, as shown in Figure 4b, we also present its SPM image. The nanostructure with a depth of 50–300 nm was formed with a single step. With the two-step process, the first step produced an interference pattern with a period of 1.9 μm, a width of 800 nm, and a depth of ~50 nm, while the second step produced a nanostructure with a period of 490 nm and a depth of ~200 nm. The lower uniformity of the depth compared to the period indicates that the ablation depth per pulse varies depending on the surface position.

## 4. Conclusions

We demonstrated the formation of a homogeneous line-like nanostructure with a period of 490 nm using the two-step ablation process with square flattop beams of femtosecond laser pulses. A nanostructure with a constant period was formed over 95% of the laser-processed area, indicating that the ratio between the nanostructure and the modification area was over six times larger than that of a Gaussian beam. The results show that combining a refractive field-mapping beam shaper and direct laser interference patterning is very simple and effective for homogeneously producing laser-induced periodic surface structures.

## Data Availability

The original contributions presented in the study are included in the article; further inquiries can be directed to the corresponding author.
